# Plant phloem sterol content: forms, putative functions, and implications for phloem-feeding insects

**DOI:** 10.3389/fpls.2013.00370

**Published:** 2013-09-24

**Authors:** Spencer T. Behmer, Nathan Olszewski, John Sebastiani, Sydney Palka, Gina Sparacino, Elizabeth Sciarrno, Robert J. Grebenok

**Affiliations:** ^1^Department of Entomology, Texas A&M University, College StationTX, USA; ^2^Department of Biology, Canisius CollegeBuffalo, NY, USA

**Keywords:** aphids, bean, cholesterol, hemiptera, insect nutritional physiology, phytosterols, tobacco

## Abstract

All eukaryotes contain sterols, which serve as structural components in cell membranes, and as precursors for important hormones. Plant vegetative tissues are known to contain mixtures of sterols, but very little is known about the sterol composition of phloem. Plants are food for many animals, but plant-feeding arthropods (including phloem-feeding insets) are unique among animals in that they have lost the ability to synthesize sterols, and must therefore acquire these essential nutrients from their food, or via endosymbionts. Our paper starts by providing a very brief overview of variation in plant sterol content, and how different sterols can affect insect herbivores, including those specializing on phloem. We then describe an experiment, where we bulk collected phloem sap exudate from bean and tobacco, and analyzed its sterol content. This approach revealed two significant observations concerning phloem sterols. First, the phloem exudate from each plant was found to contain sterols in three different fractions – free sterols, sterols conjugated to lipids (acylated), and sterols conjugated to carbohydrates (glycosylated). Second, for both plants, cholesterol was identified as the dominant sterol in each phloem exudate fraction; the remaining sterols in each fraction were a mixture of common phytosterols. We discuss our phloem exudate sterol profiles in a plant physiology/biochemistry context, and how it relates to the nutritional physiology/ecology of phloem-feeding insects. We close by proposing important next steps that will advance our knowledge concerning plant phloem sterol biology, and how phloem-sterol content might affect phloem-feeding insects.

## INTRODUCTION

**FIGURE 1:**
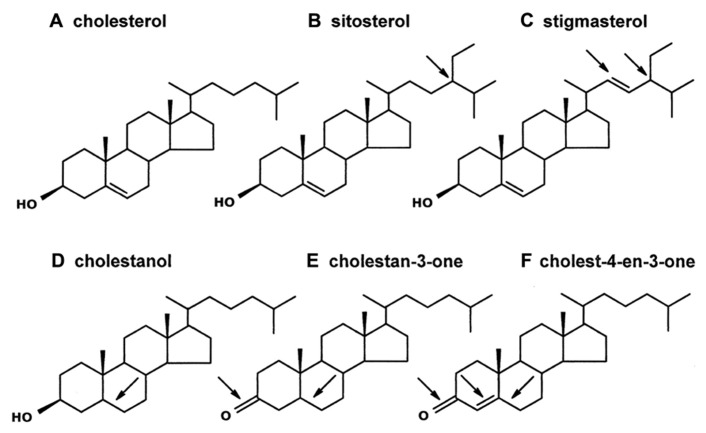
**Sterols and steroids of interest.** Cholesterol **(A)** is the common sterol in animals, including insects. Plants contain cholesterol analogs (the arrows on the remaining sterols/steroids indicate structural differences from cholesterol). Sitosterol **(B)** and stigmasterol **(C)** are two common phytosterols that each contain an ethyl group on the side-chain (at the C24-position); stigmasterol, unlike sitosterol, contains a double bond at the C22-position. The three steroids in the bottom row have been identified in a transgenic tobacco line on which aphids perform poorly (Behmer et al., 2011). Cholestanol **(D)** is similar to cholesterol but it lacks a double bond at the C5-position. Cholestan-3-one **(E)** and Cholest-4-en-3-one **(F)** both have a ketone at the C3-position, and both lack a double bond at the C5-position; cholest-4-en-3-one, in contrast to cholestan-3-one, has a double bond at the C4-position.

Sterols are found in all eukaryotes, where they serve essential roles: (1) modulating membrane permeability, fluidity, organelle identity, and protein function, (2) serving as required precursors to steroid hormones, and (3) acting as signaling molecules (Behmer and Nes, 2003; Lindsey et al., 2003; Espenshade and Hughes, 2007). Plants and animals are the most familiar eukaryotes, but they have very different sterol profiles. Cholesterol (**Figure 1A**) is the dominant sterol recovered from animals, but plants usually contain only a small amount of cholesterol. Instead, plants mostly contain phytosterols (Nes and McKean, 1977), which differ structurally from cholesterol in one or two key ways: (1) the position and extent of nuclear and side chain unsaturation and (2) the extent of C24-alkylation in the side chain (Nes and McKean, 1977; Akihisa et al., 1991). For example, the common phytosterols sitosterol and stigmasterol (but not cholesterol) have an ethyl group at the C24-position (**Figures 1B,C**); stigmasterol (but not sitosterol or cholesterol) also has a double bond at the C22-position (**Figure 1C**). Other structural differences can also occur. For instance, a double bond might be lacking in the tetracyclic nucleus (e.g., cholestanol and cholestan-3-one, **Figures 1D,E**), a ketone group, rather than a hydroxyl group, might occur at the C3-position (e.g., cholestan-3-one and cholest-4-en-3-one, **Figures 1E,F**), and/or a double bond might occur at the C4-position (e.g., cholest-4-en-3-one, **Figure 1F**). More than 100 different sterols have been identified from plant vegetative tissue, and individual plants often contain multiple sterols (Nes and McKean, 1977). It is notable, though, that plant sterol composition varies in a predictable manner with phylogeny, such that the sterol profile in plants of one family tend to be more similar to each other than to plants of different, and more distantly related families (Nes et al., 1977; Salt et al., 1991).

Although the sterol content of plant vegetative tissue has been studied intensively, little is known about the sterols in phloem sap (Forrest and Knights, 1972; Shigematsu et al., 1982; Behmer et al., 2011). Recently, Behmer et al. (2011) revealed that the sterol profile of phloem sap differs markedly from that in vegetative tissues for both Chinese cabbage and tobacco. For both plants, phytosterols (e.g., sitosterol and stigmasterol) were dominant in the vegetative tissue (Chinese cabbage = 100%; tobacco = 85%), but cholesterol was the most abundant sterol recovered in the phloem (Chinese cabbage = 40%; tobacco = 96%). This study also compared the vegetative and phloem sterol profiles of tobacco plants genetically modified to express a bacterial sterol oxidase gene. These plants are as vigorous as the wild-type controls despite producing large amounts of atypical sterol derivatives, particularly the cholesterol derivatives cholestan-3-one and cholest-4-en-3-one (Heyer et al., 2004). These two sterol derivatives were particularly concentrated in the phloem sap where they accounted for 76% of the phloem sterol content (in the vegetative tissues they represented 43% of the total sterol profile). Bouvaine et al. (2012) also showed that in the fava bean, phytosterols comprised >99% of the vegetative tissue sterol profile, but that cholesterol was the dominate sterol in the phloem (43%; sitosterol and stigmasterol were present at 17.3 and 39.4%, respectively). To date, though, all phloem sterol profile analyses have been limited to sterols that occur in the free-form; in this form they are likely transported in the aqueous phloem by a lipid-binding protein (Guelette et al., 2012). However, as in other organisms, a proportion of the sterol may be conjugated – for example, attached to a lipid (acylated), or to a carbohydrate (glycosylated; Moreau et al., 2002; Aguirre et al., 2012; Schrick et al., 2012). Acylated sterols, like free-sterols, would require a lipid-binding protein to move in the phloem. In contrast, glycosylated sterols would likely be soluble in the phloem. Unfortunately, we currently know virtually nothing about the extent to which the phloem contains sterols in free, acylated, and glycosylated forms.

Many animals, including insects, feed on plants, but all insects (in fact all arthropods) are metabolically impoverished relative to most other organisms in one crucial respect: they lack the capacity for the *de novo *synthesis of sterols (Grieneisen, 1994; Behmer and Nes, 2003). Cholesterol is the most abundant sterol recovered from most insects, including those feeding on plants (Behmer and Nes, 2003), and most insects have an absolute requirement for cholesterol as the precursor for ecdysteroid synthesis (Grieneisen, 1994). Plant-feeding insects typically generate cholesterol by metabolizing phytosterols. For example, caterpillars can readily convert sitosterol and stigmasterol to cholesterol (Svoboda and Weirich, 1995; Svoboda, 1999; Jing et al., 2012b). However, not all insects share the same sterol metabolic capabilities. Grasshoppers, for instance, cannot convert stigmasterol to cholesterol (Behmer and Elias, 1999, 2000). Other sterol metabolic constraints are more general – no plant-feeding insect can insert a double bond at the C5-position in the sterol nucleus, and there is little evidence that insects modify, other than for metabolic purposes (Brown et al., 2009), the position of double bonds in the sterol nucleus (reviewed by Behmer and Nes, 2003). When chewing insect herbivores ingest large quantities of sterols that cannot be converted into cholesterol, they often suffer reduced performance (Behmer and Grebenok, 1998; Behmer and Elias, 1999, 2000; Jing et al., 2012a, b). Additionally, unsuitable sterols can reduce feeding (Behmer et al., 1999).

Although most insects derive their sterol requirement from the diet, some insects that feed on foods of very low nutritional quality (e.g., wood) obtain sterols from yeast symbionts (Noda and Koizumi, 2003). Early studies (Ehrhardt, 1968) also implicated microbial symbionts in the sterol nutrition of plant-sap feeding insects, especially aphids, but improved analytical techniques have refuted these interpretations (Campbell and Nes, 1983). It is now appreciated that the yeast symbionts borne by a few plant sap-feeding insects (a minority of planthoppers and aphids) provide their insect hosts with sterols (Noda et al., 1979; Noda and Koizumi, 2003). In contrast, bacterial symbionts cannot provide sterols, because bacteria cannot biosynthesize sterols (Douglas, 2009). Therefore, most phloem feeding insects are dependent on the phloem sap for their sterol requirements. We have recently demonstrated that the aphid *Myzus persicae *feeds poorly and suffers high mortality on plants with the atypical sterol content, raising the hypothesis that some sterols/sterol derivatives are deleterious to the aphids (Behmer et al., 2011).

Given the sparsity of data on phloem sap sterol composition, coupled with the importance of sterols to insects specializing on phloem, the current study had two main objectives. The first was to determine the extent to which phloem sterols occur as free sterols, acylated sterols and/or steryl glycosides in two model plant systems: bean (*Phaseolus vulgaris*) and tobacco (*Nicotiana tabacum*). The second objective was to determine, for each sterol class (free, acylated, and glycosylated), the sterol composition. We discuss our findings both in the context of plant physiology, and the nutritional physiology/ecology of insects that specialize on phloem. We also propose next steps that will advance our understanding of both plant phloem sterol physiology/biochemistry, and the nutritional physiology/ecology of phloem-feeding insects.

## MATERIALS AND METHODS

### EXPERIMENTAL PLANTS

Two plant species were used: *Phaseolus vulgaris* and *Nicotiana tabacum* (var. Xanthi). Seedlings were germinated under transparent plastic drape (to maintain high humidity); upon establishing, the plastic drape was replaced with a 6-inch transparent plastic dome. When plants outgrew the plastic domes, they were grown under tents of transparent plastic drape. All plants were maintained at 23°C with 12L:12D at 120 μmol m^-^^2^ s^-^^1^ PAR; they were watered as needed and fertilized once per week.

### COLLECTION OF PHLOEM SAP

Samples for sterol analysis were obtained from multiple plant leaves (located at least 3 leaves below the apical meristem at the day of harvest) using the EDTA exudation technique developed by King and Zeevaart (1974), and later modified by Karley et al. (2002). Briefly, leaves were excised and the petioles inserted immediately into an Eppendorf tube containing 1 ml 10 mM Na^+^ EDTA solution, pH 7.5. The samples were then incubated for 60 min in the dark in a sealed chamber equilibrated at 23–25°C; the inside of the chamber was lined with water saturated paper towels to maintain high humidity.

The number of leaves harvested/plant, and the number of plant leaves used to generate each sterol exudate sample varied based on the plant. For *P. vulgaris*, each replicate sample consisted of 80 leaves (each cut at the base of the petiole (near the stem) using a sterile, sharp #10 scalpel). Specifically, 10 individual Eppendorf tubes, each containing 1 ml of EDTA solution, plus 8 leaves, were established. After the 1 h incubation period, the 10 individual EDTA exudate solutions were consolidated into a single 10 ml sample (held in a 20 ml borosilicate glass vial, in the refrigerator, until used for sterol analysis). For *N. tabacum*, there was only one leaf/Eppendorf tube, but as above a replicate consisted of 10 consolidated EDTA solutions. Bulking the samples in this way provided a sufficient amount of phloem exudate on which sterol analysis could be performed.

### STEROL ANALYSIS OF PHLOEM SAP EXUDATE

Analysis on three different sterol forms was conducted: (1) “free” (unbound 3′ hydroxyl group), (2) “acylated” (bound 3′ hydroxyl group, typically lipid bound), and (3) “glycosylated” (bound 3′ hydroxyl group, typically carbohydrate bound). The number of replicates for each sterol form, and for each plant, ranged from 3 to 6 (see **Table 1**).

**Table 1:** Phloem sap sterol content for *Phaseolus vulgaris* and *Nicotiana tabacum*.

Plant	Median total phloem sterol amount (μg)	Sterol content [adjusted median (w/w) of total in each sterol form]	
		Cholesterol	Sitosterol	Campesterol	Stigmasterol
**(a) *Phaseolus vulgaris***
Free sterols (4)	28.0	100.0	-	-	-
Acylated sterols (5)	60.1	100.0	-	-	-
Glycosylated sterols (4)	130.8	100.0	-	-	-
**(b) *Nicotiana tabacum***
Free sterols (3)	761.3	97.8	0.9	-	1.3
Acylated sterols (6)	351.7	98.2	1.7	-	0.1
Glycosylated sterols (3)	1026.6	88.3	-	11.7	-

*For each plant three different sterol forms were collected: (i) free, (ii) acylated (lipid conjugated) and (iii) glycoslyated (sugar conjugated). The number of replicate samples for each sterol form, from each plant, is indicated in parentheses.*

The first step for each sterol sample, regardless of form being analyzed, was the addition 5 ml of 100% MeOH (pre-equilibrated to hexane), plus 5 ml 100% hexane (pre-equilibrated to 50% MeoH/water). Additionally, 10 μg of cholestane was added to each sample (this served as an internal standard). Next, each sample was shaken vigorously for several seconds, followed by incubation at room temperature for 24 h in the dark. The hexane fraction (containing free and acylated sterols) was then separated from the MeOH/water fraction (containing the glycosylated sterols), and both fractions were evaporated to dryness using nitrogen. For each sample, the hexane fraction was processed further for quantification of either the free sterols or acylated sterols, while each MeOH/water fraction was processed further for quantification of glycosylated sterols.

For free sterol analysis, 50% of the hexane fraction was taken, conjugated, and analyzed by GC–MS. For acylated sterol analysis, the remaining 50% of the hexane fraction was resuspended in 100 μl of clean hexane and 8 ml of 70% MeOH-water containing 5% KOH was added, and then incubated in a shaking water bath (225 rpm) at 55°C for 2.5 h. This replaces the lipid moiety at C3 with a free hydroxyl group. The MeOH-water fractions were resuspended in 8 ml 100% methanol containing 10% HCl, and then incubated in a shaking water bath (225 rpm) at 55°C for 2.5 h, to remove the carbohydrate moiety present at C3; it was replaced with a free hydroxyl group. Subsequently, all fractions contained free sterols, which were extracted from the chemically treated samples with water-equilibrated hexane; the hexane layer was then washed to neutrality with hexane-equilibrated water. The recovery rate of our internal standard (cholestane) was 92 ± 5%. The level of detection for GC–MS was tens of nanograms; detection at this low level was made possible using selected ion chromatogram software, and selected ion monitoring software [GC–MSD ChemStation (Agilent Technologies)].

The sterols contained in the three fractions were converted to their respective trimethylsilyl ether (TMS) deriviatives, to ensure the inertness of the free C3 hydroxyl, by overnight incubation with a 2:1 excess volume v/v of BSTFA + TMCS, 99:1 (Sylon BFT; Supelco Inc. Bellefonte, PA, USA). All conjugated sterols were processed by gas chromatography – mass spectroscopy (GC–MS), using an Agilent 6850N GC coupled with a 5973 mass selective detector (Agilent Technologies, Inc., Santa Clara, CA, USA). The GC–MS was equipped with a fused capillary EC-5 column (30 m; Alltech, Nicholasville, KY, USA) with a 0.25 mm internal diameter and 0.25 μm film thickness. The running conditions were: inlet 280°C, transfer line 290°C, column 80°C (1 min), ramp at 10°C min^-^^1^ to 240°C, 240 to 300°C, ramp of 5°C min^-^^1^, with helium (1.2 ml min^-^^1^) as carrier gas. The Agilent 5973 mass selective detector maintained an ion source at 250°C and quadrupole at 180°C. Sterols were identified and quantified by GC–MS using selected ion monitoring (SIM) protocols for each steroid identified (Rahier and Benveniste, 1989). Authentic sterol standards were purchased commercially [from Sigma Chemical (St. Louis, MO, USA), and Steraloids Inc. (Newport, RI, USA)].****

## RESULTS

Phloem sap was collected from two different plant species (*P. vulgaris* and *N. tabacum*), and for each plant we identified and quantified the abundance of free, acylated and glycosylated sterols. Both plants contained sterols in all three forms (**Table 1**), but the amounts of each sterol form recovered from the phloem sap differed between the two plant species. For example, *P. vulgaris* contained large pools of glycosylated sterols, intermediate pools of acylated sterols, and relatively small pools of free sterols. In *N. tabacum*, glycosylated sterols were again the largest sterol pool, but in contrast to *P. vulgaris*, *N. tabacum* contained free sterols in rather large amounts, while acylated sterols were the smallest pool.

For both plant species, cholesterol dominated each of the sterol fractions (**Table 1**). For *P. vulgaris*, cholesterol was the only sterol recovered from the glycosylated-sterol fraction, and the only sterol recovered from 3 of the 4 free-sterol fractions, and from 4 of the 5 acylated-sterol fractions. A single free-sterol sample contained cholesterol and sitosterol (87 and 13%, respectively), and a single acylated-sterol sample contained cholesterol, sitosterol and campesterol (53, 28 and 19%, respectively). In the case of *N. tabacum*, the sterol fractions generally contained multiple sterols (2–4), but cholesterol was always the most abundant. There were, however, some trends with respect to the non-cholesterol sterol profiles of the different tobacco sterol fractions. Generally, small amounts of sitosterol and stigmasterol, but not campesterol, were recovered in both the free- and acylated-sterol fractions. In contrast, campesterol, but not sitosterol or stigmasterol, was recovered in the glycosylated-sterol fraction.

## DISCUSSION

The collection of phloem sap in the presence of EDTA is a standard technique for isolating phloem sap from whole plants (King and Zeevaart, 1974; Costello et al., 1982; Karley et al., 2002). Madey et al. (2002) used this technique to isolate and characterize the lipids found in canola phloem sap, and confirmed that lipid exudates collected in the presence of EDTA originated from the phloem, not the xylem. To date, the presence of phytosterols in the phloem of higher plants has been demonstrated both indirectly (e.g., from aphid honeydew; Behmer et al., 2011), and directly (e.g., via phloem sap collection in the presences of EDTA (Behmer et al., 2011; Bouvaine et al., 2012), as has the capacity for the movement of sterols within the phloem (Grebenok and Adler, 1993). However, in each of these instances the focus was entirely on free sterols, for two primary reasons. First, in the case of phloem and honeydew, only small amounts of material could be collected, which precluded a more thorough investigation into the presence of conjugated sterols. Second, with respect to sterol movement in the phloem, analysis for sterol conjugates was simply not considered. In the current study we employed a bulk collection technique, where petioles of multiple leaves were placed in EDTA containing solutions, to ensure that a sufficient quantity of phloem sap could be collected for analysis of sterol form, as well as sterol composition. Our data demonstrate, for the first time, that sterols in the phloem of higher plants are maintained as free sterols, acylated sterols and sterol glycosides. Additionally, our results show that the percentage of these different sterol-forms can vary between plants, and that cholesterol was the most abundant sterol for each sterol fraction.

On average, about half of the phloem sterol pool (47–60%) was glycosylated; the remaining half of sterol was a mixture of free- and acylated forms. Considering that the phloem stream is aqueous, it perhaps should not be surprising to find relatively large pools of glycosylated sterol in the phloem. Glycosylation makes sense, as it affords sterols greater solubility in the phloem. Sterols are known to be important signaling molecules, in both animals (Incardona and Eaton, 2000) and plants (Clouse, 2002), and given the description of the phloem acting as an “information superhighway” in plants (Lucas and Wolf, 1999), sterols in a glycosylated form could move relatively unencumbered in the phloem, and in this form interact more readily with potential receptor sites on individual cells (in a fashion similar to hormones, which also operate at low concentrations). On the other hand, movement of free- and acylated-sterols would require a shuttle system. Animals, including insects, use a protein shuttle system to transport sterols in their blood (Arrese and Loulages, 2010), and although putative proteins that provide such a role have been recently identified in plants (Guelette et al., 2012), no data exist to link the association of sterols with a phloem resident protein. Alternatively, free and acylated sterols may form, with other lipids, mixed micelle particles, with their hydrophilic elements oriented outward, and their hydrophobic elements oriented inwards. Madey et al. (2002) found that phloem lipid particles in canola phloem sap were organized in the form of spherical particles, which were of variable size. Such particles might also facilitate bulk transport of sterols, over relatively long distance, where they might serve a developmental or physiological function, for instance in cells in membranes (Demel and De kruyff, 1976), as promoters of cell division (Haughan et al., 1988), or for sensing osmotic change (Zelazny et al., 1995).

Perhaps the most notable result is the extent to which cholesterol was the dominant sterol recovered, from each of the three sterol fractions. This is in complete contrast to what is typically observed in vegetative tissues (Nes and McKean, 1977; Nes et al., 1977; Salt et al., 1991). But given that cholesterol is only a miniscule percentage of a typical vegetative tissue sterol profile (Nes et al., 1977; Salt et al., 1991; Behmer et al., 2011), why is it the dominant sterol within the phloem stream? If cholesterol in the phloem is not acting as a signaling molecule, or providing direct physiological function, perhaps it is present as a precursor to important plant steroid hormones, such as brassinosteroid. Insects require very small pools of cholesterol as the precursor to the steroid molting hormones (Gilbert et al., 2002), and in plants cholesterol is a close analog to 24-methylene cholesterol (Benveniste, 2002), which is an intermediate in the synthesis of campesterol, the direct precursor to brassinosteroid (Noguchi et al., 1999). Alternatively, the different chemical forms of cholesterol in the phloem stream might be associating with different targets, potentially facilitating multiple outcomes. There is also a question of where phloem sterols originate. Devarenne et al. (2002) demonstrated that sterol biosynthetic enzymes are associated with the phloem, and suggested that sterols can be synthesized in the phloem. Considering the presence of both sterol biosynthetic enzymes and cholesterol in the phloem, it is also possible that structural sterols are synthesized in the phloem and transported where they are needed (Devarenne et al., 2002). Although these functions need not be mutually exclusive, experiments focused on the growth, development and physiological response of the plant to various abiotic factors (e.g., temperature extremes, light conditions, humidity levels and volatile chemicals), coupled with the collection of phloem exudate from select leaves, will help clarify these relationships.

For phloem-feeding insects, which perform best on diets containing cholesterol (Bouvaine et al., 2012), and which have low dealkylation efficiency (Campbell and Nes, 1983), having access to a food source rich in cholesterol is very advantageous. However, given that a high proportion of sterols in the phloem can be in a conjugated form, it will be important to experimentally test how available these sterols are to phloem-feeding insects, such as aphids. At a minimum, phloem-feeding insects would require active glucosidases and/or acylases in the midgut. Sterol form may also have implications related to experiments using chemically-defined diets. One intriguing aspect about aphids reared on chemically-defined diets is that performance relative to plants is often greatly reduced, especially with respect to reproduction (Douglas, 2003). The chemically-defined diets typically used in these studies are presented to aphids as aqueous solutions contained within a Parafilm packet/sachet (Kunkel, 1976; Douglas, 1988; Bouvaine et al., 2012), with the sterols either solubilized, or incorporated into liposomes, and then added to the aqueous solution. Unfortunately we know little about how evenly sterols, delivered using either of these methods, are distributed in the chemically-defined diet, or the extent to which they are ingested. Knowing that phloem sap contains a significant proportion of glycosylated sterols, it would be illuminating to reevaluate aphid performance on diets containing free-, acylated-, and glycosylated cholesterol. A simple hypothesis is that performance would be superior on diets containing glycosylated sterols.

The data we present, and the story we tell, is in many ways very preliminary as we have no information on the identity of the acylated/glycosylated groups attached to phloem sterols, no information on the absolute amounts of each different sterol form and type present in the phloem, and no clear understanding of what biotic or abiotic factors influence the level and types of sterols present in the phloem sap. Additionally, while our collective data show that the sterol profile of the phloem is dominated by cholesterol, we still see phloem sterol profile differences between closely related plants (e.g., the common bean and fava bean). Another key outstanding issue is the extent to which cholesterol is the dominant phloem sap sterol in all plants. For instance, all the plants so far studied (with respect to phloem sap sterols) accumulate Δ^5^-sterols, but some plants accumulate Δ^7^-sterols in the vegetative tissue (e.g., spinach and *Solidago*). In these plants is cholesterol (a Δ^5^-sterol) the dominant phloem sap sterol? Unpublished preliminary data involving spinach, and sterol profiles from hemipterans feeding on *Solidago* (Janson et al., 2009), suggest this might be the case. Comparative studies, sampling phloem sap exudate from a variety of taxonomically diverse plants, and combined with sterol tissue analysis of phloem feeding insects (work currently underway), will help shed light on the extent to which cholesterol is widely occurring, and the dominant phloem sap sterol in plants. Such studies will also provide insight into the potential of modifying plant sterol profiles as a novel way to manage hemipteran pest populations (Behmer et al., 2011).

## Conflict of Interest Statement

The authors declare that the research was conducted in the absence of any commercial or financial relationships that could be construed as a potential conflict of interest.
